# The Role of RANBP3L in Pan‐Cancer With Its Significance in Hepatocellular Carcinoma

**DOI:** 10.1002/cnr2.70440

**Published:** 2025-12-29

**Authors:** Beini Cen, Jie Li, Chao Wang

**Affiliations:** ^1^ Zhejiang Key Laboratory of Zero Magnetic Medicine, Affiliated Hangzhou First People's Hospital, School of Medicine Westlake University Hangzhou China; ^2^ Department of Hepatobiliary and Pancreatic Surgery, Affiliated Hangzhou First People's Hospital, Westlake University School of Medicine Westlake University Hangzhou China

**Keywords:** biomarkers, liver hepatocellular carcinoma, RANBP3L, TCGA

## Abstract

**Background:**

RAN binding protein 3‐like (RANBP3L), a member of the Ran‐binding protein family, has been linked to various cellular functions, but the role in cancer remains underexplored. In this research, we assessed the diagnostic and prognostic value of RANBP3L in pan‐cancer, especially in liver hepatocellular carcinoma (LIHC).

**Aims:**

This study aimed to explore the pan‐cancer expression, prognostic value, and immune‐related roles of RANBP3L, especially emphasis on validating its diagnostic and prognostic significance in hepatocellular carcinoma.

**Methods:**

We analyzed RANBP3L expression of 33 cancer types from TCGA data and assessed its relationship with overall survival (OS), disease‐specific survival (DSS), and progression‐free interval (PFI). We used TIMER2.0 and CIBERSORT to explore the correlation between RANBP3L expression and immune cells. We conducted immunohistochemistry, qRT‐PCR, and western blotting by using tissue samples from LIHC patients to assess the RANBP3L's diagnostic and prognostic value of LIHC.

**Results:**

In 18 different cancers, RANBP3L expression was found to be lower in tumor tissues compared to normal tissues, including LIHC. Lower RANBP3L expression was related to shorter OS, DSS, and PFI in LIHC. ROC analysis and the nomogram model based on RANBP3L expression demonstrated high predictive accuracy for patient diagnosis and survival. Moreover, immune infiltration analysis showed that RANBP3L was related to various immune cells and impacted prognosis. Furthermore, analysis of LIHC patient tissues found that higher RANBP3L expression was related to better tumor‐free survival and OS.

**Conclusion:**

RANBP3L plays a crucial role in LIHC. Not only does its expression level correlate with patient survival, but it also plays an important role in immune modulation. RANBP3L presents a promising candidate for future therapeutic strategies and a biomarker for LIHC.

## 1 Introduction

Cancer is an important global health challenge, with rising incidence and mortality rates each year [1]. Among those prevalent cancers, liver hepatocellular carcinoma (LIHC) is particularly associated with high mortality rates worldwide [2]. Despite ongoing advancements in cancer diagnosis and treatment, the five‐year overall survival (OS) rates for many cancers remain low, highlighting the need for more effective therapeutic strategies [3]. While substantial progress has been made in utilizing cancer biomarkers for diagnosis and prognosis in certain cancers [4], the lack of effective biomarkers for liver and other cancers remains a significant barrier, given the need for innovative diagnostic and therapeutic methods to improve patient outcomes and offer new avenues for treatment [5].

RAN binding protein 3 like (RANBP3L) is from the family of Ran‐binding proteins, which featured the presence of the Ran‐binding domain [6]. Ran (Ras‐related nuclear protein) is a small GTPase from the Ras superfamily, which could regulate nucleocytoplasmic transport of molecules and control cell cycle progression [7]. It has been reported that RANBP3L could modulate bone morphogenetic protein signaling and the mesenchymal stem cells differentiation [8]. There are little researches about RANBP3L in tumors, only one report shows that the immune cell enrichment score of RANBP3L combined with other seven genes can predict outcomes in endometrial cancer patients [9].

This research was to explore the RANBP3L expression and function in multiple cancers by database, especially in LIHC. Then we assessed the relationship between the RANBP3L expression and immune cell infiltration. Finally, we utilized LIHC samples to investigate the RANBP3L expression and prognostic value.

## 2 Methods

### 2.1 Database‐Driven Analysis

The expression of RANBP3L in the 33 cancers (Table 1) was obtained from the Cancer Genome Atlas (TCGA) database (https://portal.gdc.cancer.gov/). The clinical data came from the TCGA database.

**TABLE 1:** The abbreviation of 33 cancers.

Abbreviation	Type of cancer
ACC	Adrenocortical carcinoma
BLCA	Bladder urothelial carcinoma
BRCA	Breast invasive carcinoma
CESC	Cervical and endocervical cancers
CHOL	Cholangiocarcinoma
COAD	Colon adenocarcinoma
DLBC	Diffuse large B‐cell lymphoma
ESCA	Esophageal carcinoma
GBM	Glioblastoma multiforme
HNSC	Head and neck squamous cell carcinoma
KICH	Kidney chromophobe
KIRC	Kidney renal clear cell carcinoma
KIRP	Kidney renal papillary cell carcinoma
LAML	Acute myeloid leukemia
LGG	Brain lower grade glioma
LIHC	Liver hepatocellular carcinoma
LUAD	Lung adenocarcinoma
LUSC	Lung squamous cell carcinoma
MESO	Mesothelioma
OV	Ovarian serous cystadenocarcinoma
PAAD	Pancreatic adenocarcinoma
PCPG	Pheochromocytoma and paraganglioma
PRAD	Prostate adenocarcinoma
READ	Rectum adenocarcinoma
SARC	Sarcoma
SKCM	Skin cutaneous melanoma
STAD	Stomach adenocarcinoma
TGCT	Testicular germ cell tumors
THCA	Thyroid carcinoma
THYM	Thymoma
UCEC	Uterine corpus endometrial carcinoma
UCS	Uterine carcinosarcoma
UVM	Uveal melanoma

Kaplan–Meier analysis with log‐rank test was used to analyze prognosis included OS (overall survival), DSS (disease‐specific survival) and PFI (progression‐free interval). For cancers the RANBP3L expression was linked to the prognosis, the association between clinical characteristic and RANBP3L expression was explored. We used Spearman rank test and Wilcoxon rank‐sum test for clinical characteristic analysis. The ROC analysis was conducted applying the “pROC” package. The nomogram model of RANBP3L in LIHC was established according to the RANBP3L expression and the clinical stage to predict the OS. The calibration curve was used to evaluate the predictive accuracy of the nomograms for 1‐year, 3‐year, and 5‐year survival outcomes.

We used TIMER2.0 (Tumor Immune Estimation Resource 2.0, http://timer.cistrome.org/) [10] to explore the correlation between RANBP3L expression and the immune cell infiltration of various tumors in TCGA. Multiple algorithms, including TIMER2.0 and CIBERSORT (https://cibersortx.stanford.edu/) [11], were applied for the immune cell estimation. Additionally, we explored the correlation of immune cell infiltration and OS of RANBP3L expression across different cancer types.

We draw the protein–protein interaction (PPI) network according to the STRING data (https://string‐db.org/), with the interaction threshold of 0.4. The Gene Ontology (GO) analysis contains the biological pathways (BP), the molecular functions (MF), and the cellular components (CC). The Kyoto Encyclopedia of Genes and Genomes (KEGG) analysis was also performed meanwhile. The clusterProfiler R package and the DESeq R package were used for GESA in this study.

### 2.2 Patients and Samples Collection

Tissue microarrays were constructed using 70 LIHC tissue samples which were got from patients that did hepatectomy at the Hangzhou First People's Hospital. Another 6 paired HCC tissues and the para‐carcinoma tissues used for western blot and RT‐qPCR were got from patients who did hepatectomy at Hangzhou First People's Hospital. Ethical Committee approved the research, and each patient signed the informed consent form. This research was carried out following the ethical principles outlined in the Declaration of Helsinki. The study was approved by the Institutional Ethics Committee of Hangzhou First People's Hospital (Approval No. 2022‐MedEthics‐384). The diagnosis of HCC was confirmed by pathological examination for every patient. The para‐carcinoma tissue was defined as tissue at least 1 cm away from the margin of the carcinoma tissue.

### 2.3 Quantitative Real‐Time Polymerase Chain Reaction (qRT‐PCR)

The RNA extraction specific experimental steps could refer to this literature [12]. GAPDH was applied as an internal standard. The following primer sequences were used: RANBP3L: Forward Primer (5′‐3′), AAATCTGTCATTGCTCAACCCA and Reverse Primer (5′‐3′), GCTGCTTCATACAGGGTGTCTT; GAPDH: Forward Primer (5′‐3′), GAACGGGAAGCTCACTGG, and Reverse Primer (5′‐3′), GCCTGCTTCACCACCTTCT. The results were calculated by the 2^−ΔΔCt^ method, with each sample being tested in triplicate.

### 2.4 Western Blot (WB)

The extraction of protein and the specific experimental steps could refer to this article [13]. TTC39A Polyclonal antibody (No. 17875‐1‐AP) was purchased from Proteintech Group Inc. GAPDH (No. 60004‐1‐Ig), which was used as an internal standard, was also purchased from Proteintech Group Inc. Immunodetection was performed by an enhanced chemiluminescence (ECL) detection kit. Then the grayscale values were calculated by the Image J software.

### 2.5 Immunohistochemistry

Immunohistochemistry was performed with the tissue microarrays, made by the 70 pairs of LIHC tissues and the para‐carcinoma tissues. The specific experimental steps could refer to this literature [13]. TTC39A Polyclonal antibody (No. 21323‐1‐AP) was purchased from Proteintech Group Inc. The H‐Score was used to estimate the results of immunohistochemistry, using the Visiopharm software. The H‐score ranged from 0 to 300; the higher values mean stronger overall positive intensity [14].

## 3 Results

### 3.1 Comparison of RANBP3L Expression Between Normal Tissues and Cancer Tissues

**FIGURE 1:**
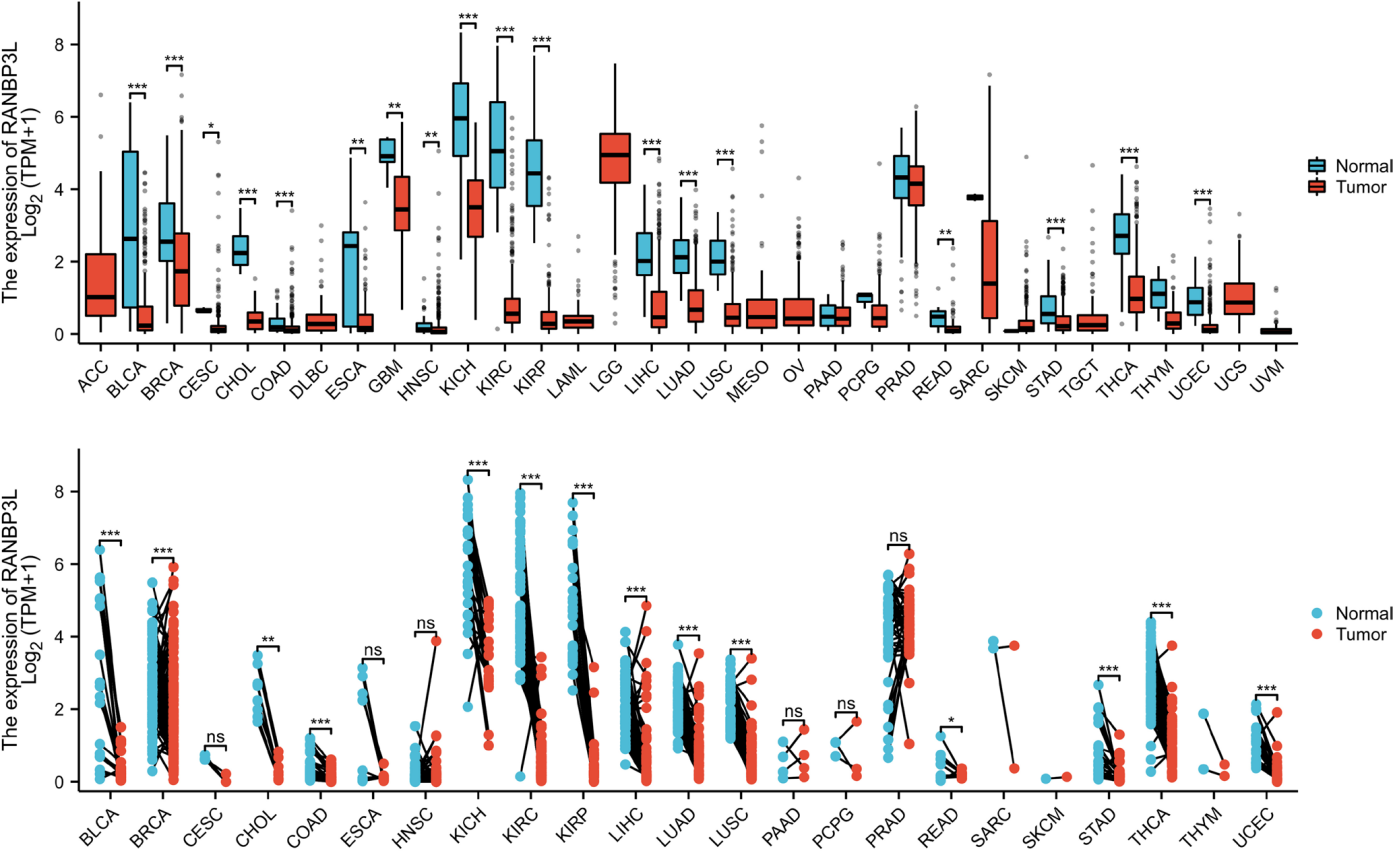
Comparison of RANBP3L expression between cancer tissues and normal tissues. (A) Comparison of RANBP3L expression between cancer tissues and normal tissues in un‐paired samples. (B) Comparison of RANBP3L expression between cancer tissues and normal tissues in paired samples. T test was used, *p < 0.05, **p < 0.01, ***p < 0.001, ns: Not significant.

RANBP3L expression for un‐paired samples was displayed in 33 cancers according to the TCGA database. Among these 33 cancers, RANBP3L expression was reduced in tumor tissues compared to normal tissues in 18 cancers, including LIHC, KIRC, LUAD, and so on (Figure 1A). Similarly, the expression of RANBP3L for paired samples was also found in 23 cancers according to the TCGA database. Among these 23 cancers, RANBP3L expression was reduced in tumor tissues compared to normal tissues in 14 cancers, including LIHC, KIRC, LUAD, and so on (Figure 1B).

### 3.2 RANBP3L Expression Was Correlated With the Patients' Prognosis

**FIGURE 2:**
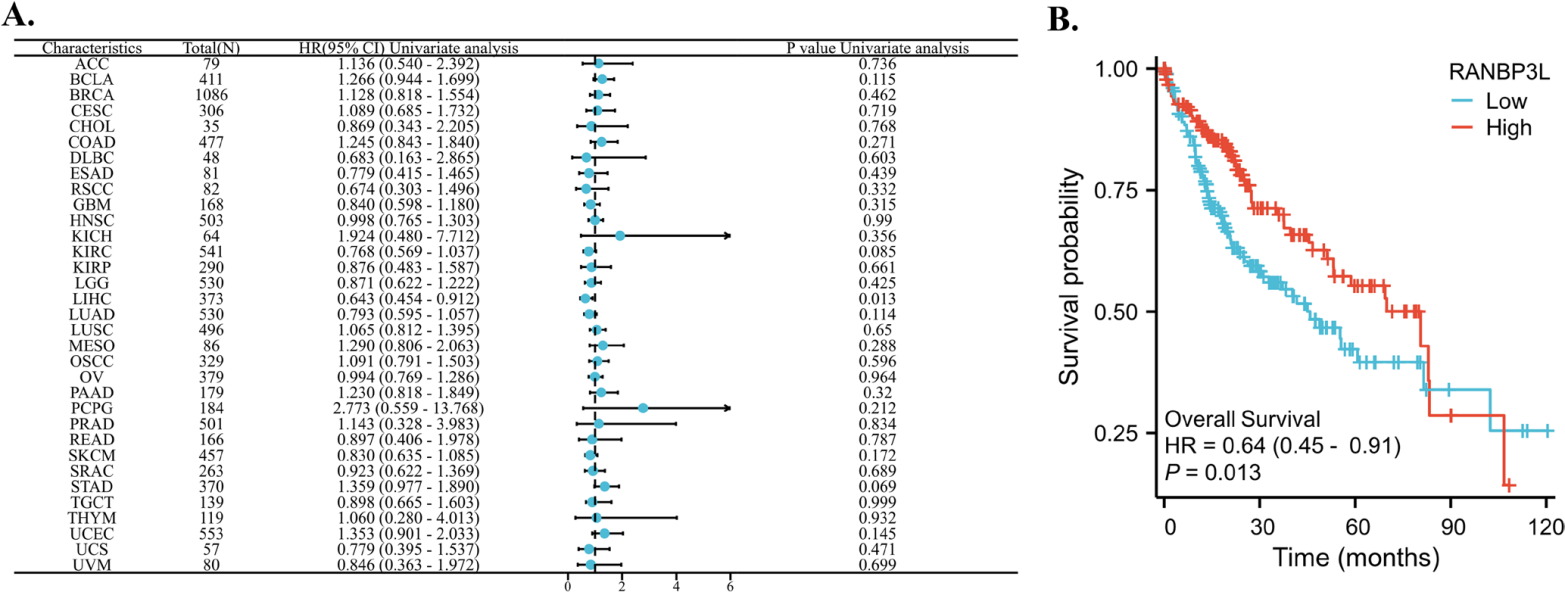
The relationship between the expression of RANBP3L and the OS of patients with 33 cancers, respectively. (A) a forest map, which showed the relationship between the expression of RANBP3L and the OS of patients for 33 cancers, respectively. (B) the expression of RANBP3L was significantly associated with the OS of patients with LIHC. *p* < 0.05 was considered significant.

The RANBP3L expression was linked to patients' OS with LIHC (Figure 2A). For LIHC patients, the lower expression of RANBP3L in cancer tissues indicated worse OS, respectively (Figure 2B).

**FIGURE 3:**
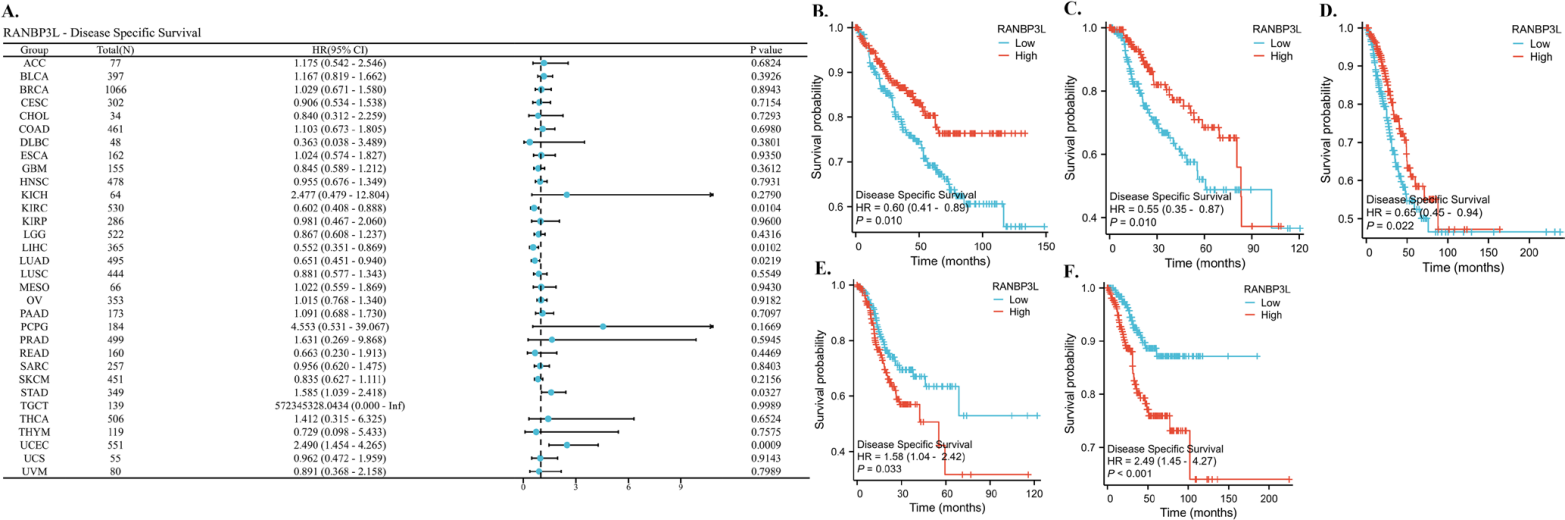
The relationship between the expression of RANBP3L and the DSS of patients with 33 cancers, respectively. (A) a forest map, which showed the relationship between the expression of RANBP3L and the DSS of patients for 33 cancers, respectively. (B–F) the expression of RANBP3L was significantly associated with the DSS of patients with KIRC, LIHC, LUAD, STAD, and UCEC, respectively. *p* < 0.05 was considered significant.

The RANBP3L expression was correlated with patients' DSS with KIRC, LIHC, LUAD, STAD, and UCEC (Figure 3A). For KIRC, LIHC and LUAD, lower RANBP3L expression in cancer tissues was associated with poorer DSS (Figure 3B–D). For STAD and UCEC, the higher RANBP3L expression in cancer tissues indicated worse DSS, respectively (Figure 3E,F).

**FIGURE 4:**
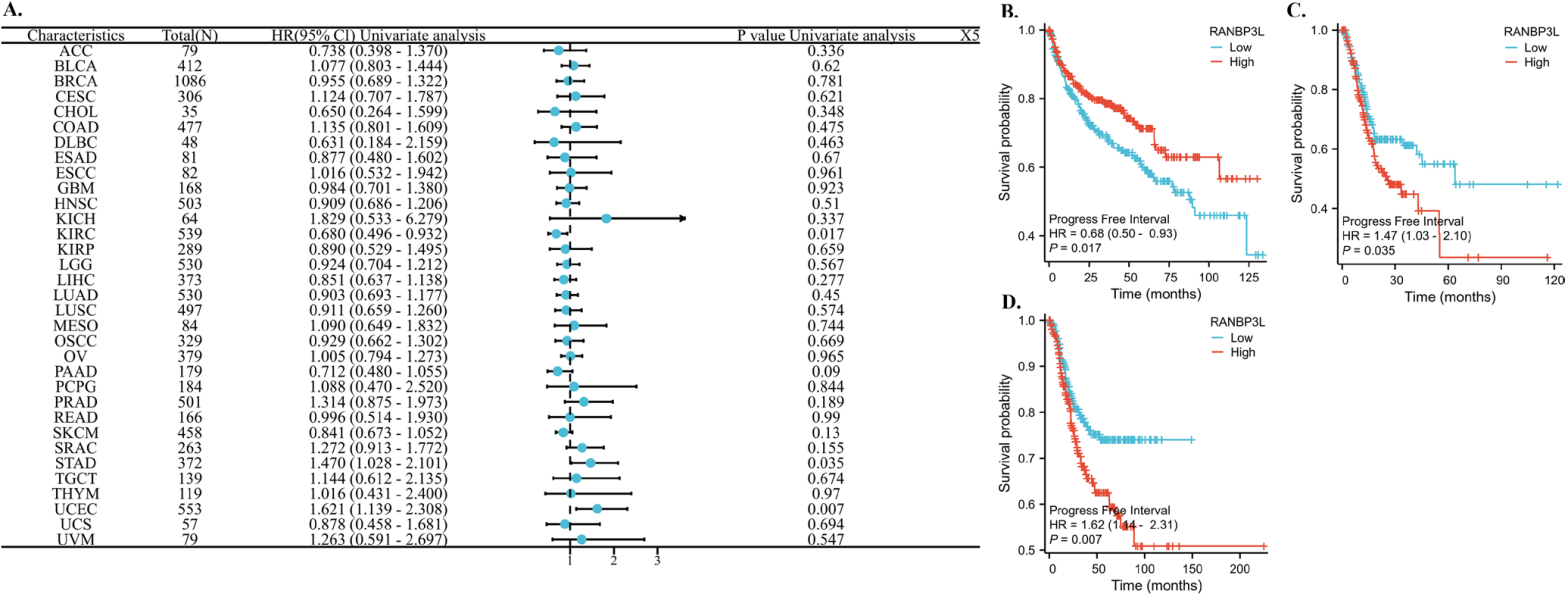
The relationship between the expression of RANBP3L and the PFI of patients with 33 cancers, respectively. (A) a forest map, which showed the relationship between the expression of RANBP3L and the PFI of patients for 33 cancers, respectively. (B–D), the expression of RANBP3L was significantly associated with the PFI of patients with KIRC, STAD, and UCEC, respectively. *p* < 0.05 was considered significant.

The RANBP3L expression was correlated with patients' PFI with KIRC, STAD and UCEC (Figure 4A). For KIRC, the lower RANBP3L expression in cancer tissues means worse PFI (Figure 4B). For STAD and UCEC, the higher RANBP3L expression in cancer tissues means worse PFI (Figure 4C,D).

### 3.3 RANBP3L Expression Was Correlated With Patients' Clinical Features

**FIGURE 5:**
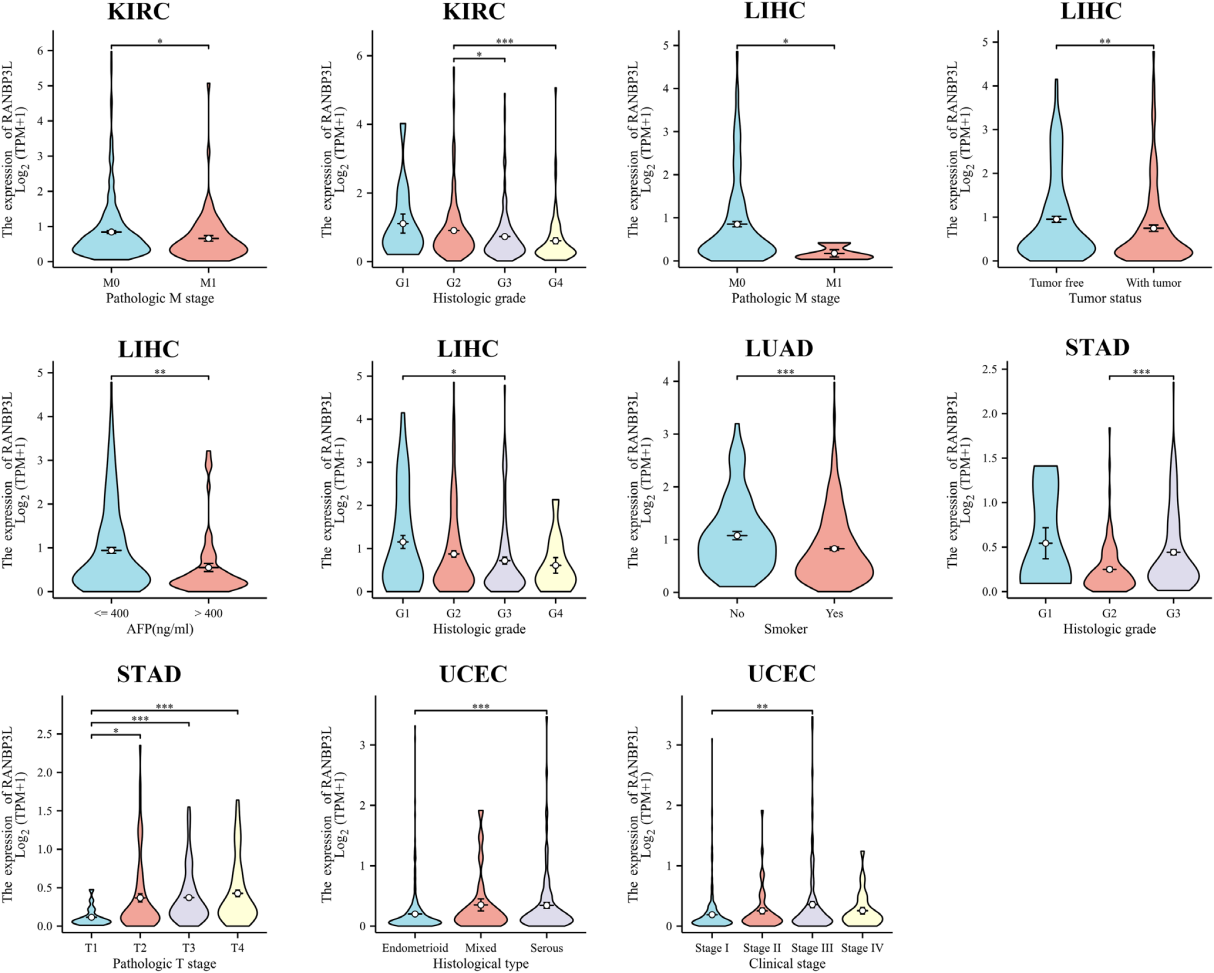
The expression of RANBP3L was associated with some clinical characteristics for patients with KIRC, LIHC, LUAD, STAD, and UCEC, respectively. *p < 0.05, **p < 0.01, ***p < 0.001.

For cancers that the RANBP3L expression was related to patients' prognosis, the connection between RANBP3L expression and patients' clinical profiles was further analyzed, according to the TCGA database (Figure 5). In KIRC, the RANBP3L expression was related to pathologic M stage and histologic grade. The RANBP3L expression was significantly related to pathologic M stage, tumor status, AFP (ng/mL), and histologic grade in LIHC. In LUAD, the RANBP3L expression was significantly linked with smoking. In STAD, the RANBP3L expression was significantly related to pathological grade and pathologic T stage. In UCEC, the RANBP3L expression was significantly linked with histological type and clinical stage.

### 3.4 The ROC Curve and Nomogram Model According to the Expression of RANBP3L in LIHC

**FIGURE 6:**
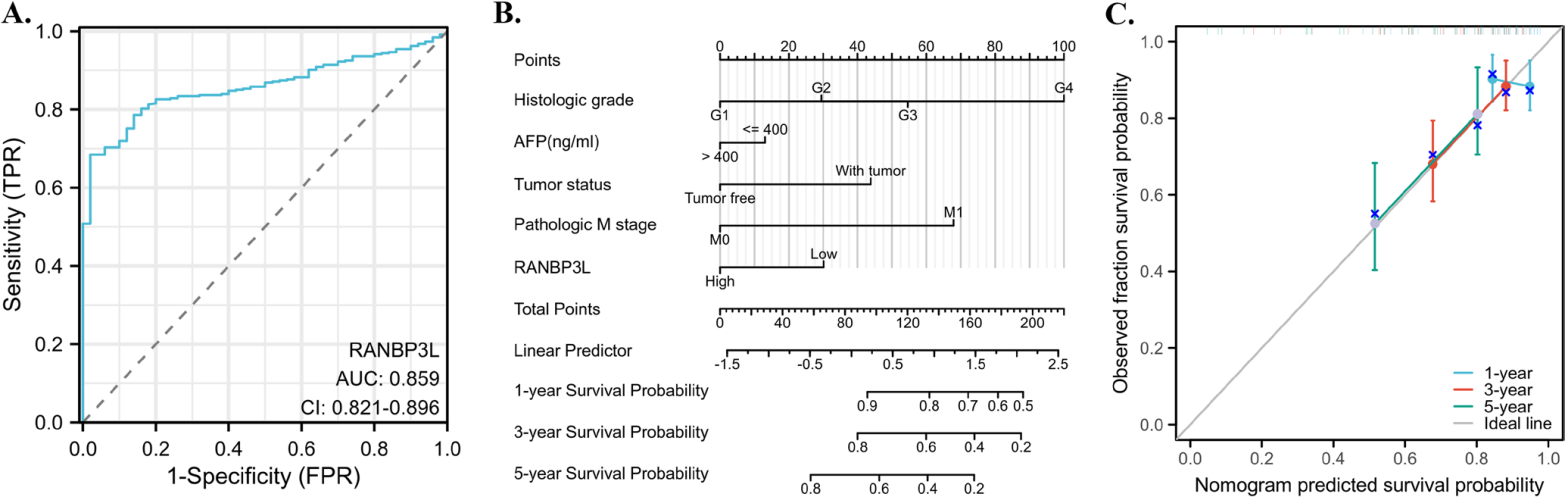
Diagnostic and prognostic effect of RANBP3L in LIHC. (A) RANBP3L could be a good diagnostic indicator for LIHC patients. (B) and (C) RANBP3L could accurately predict the 1‐year, 3‐year, and 5‐year survival probability for LIHC patients.

Among the 33 cancers, the RANBP3L expression was linked to the LIHC patients' OS and DSS and further correlated with AFP levels and pathological grade. Therefore, we investigated the diagnostic and prognostic value of RANBP3L specifically in this cancer type, for which ROC analysis and a nomogram model were subsequently established based on its expression in LIHC. The ROC analysis showed RANBP3L could be a diagnostic indicator for LIHC, with an Area Under Curve about 0.859 (Figure 6A). The nomogram model demonstrated high precision in estimating the 1‐year, 3‐year, and 5‐year survival probabilities for LIHC patients, as evidenced by the well‐calibrated survival prediction curves for these time points (Figure 6B,C).

### 3.5 The RANBP3L Expression Was Associated With the Immune Cells Infiltration and Could Affect the LIHC Patients' OS Combined With the Immune Infiltration Score

**FIGURE 7:**
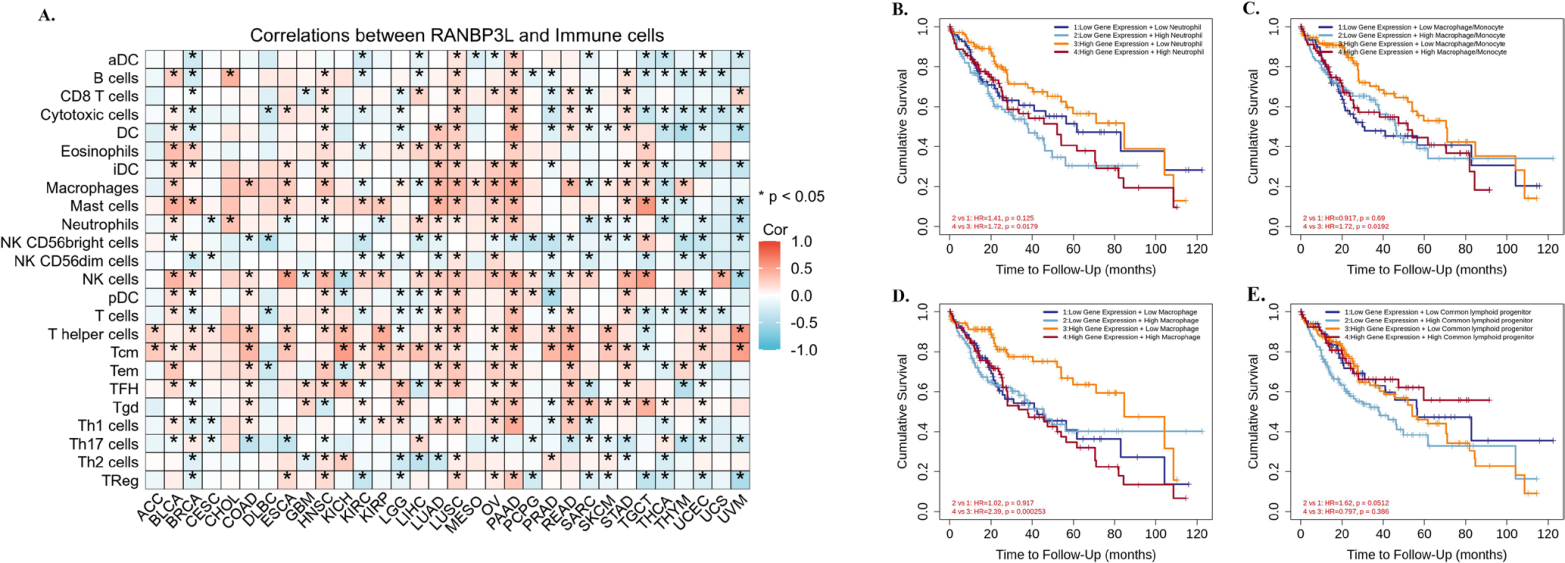
(A) Relationship between RANBP3L expression and immune cell infiltration in different cancers. *p < 0.05. The effect of immune cell infiltration on OS was related to the expression of RANBP3L. (B–E) Effect of neutrophils, macrophage, macrophage/monocyte, and common lymphoid progenitor infiltration on OS of LIHC at different RANBP3L expression levels.

CIBERSORT analysis showed that in some cancer types, a relationship was observed between the levels of immune cell infiltration and RANBP3L expression (Figure 7). We observed that RANBP3L expression showed the strongest positive correlations with immunosuppressive cells such as M2 macrophages, regulatory T cells, and dendritic cells. RANBP3L expression had a relation with infiltrating B cells, CD8 T cells, macrophages, neutrophils, NK cells, pDC, T cells, Tcm, TFH, Th17 cells, and Th2 cells in LIHC. Neutrophils, macrophage, macrophage/monocyte, and common lymphoid progenitor infiltration was related to OS in LIHC.

### 3.6 The Potential Functions of RANBP3L in LIHC

**FIGURE 8:**
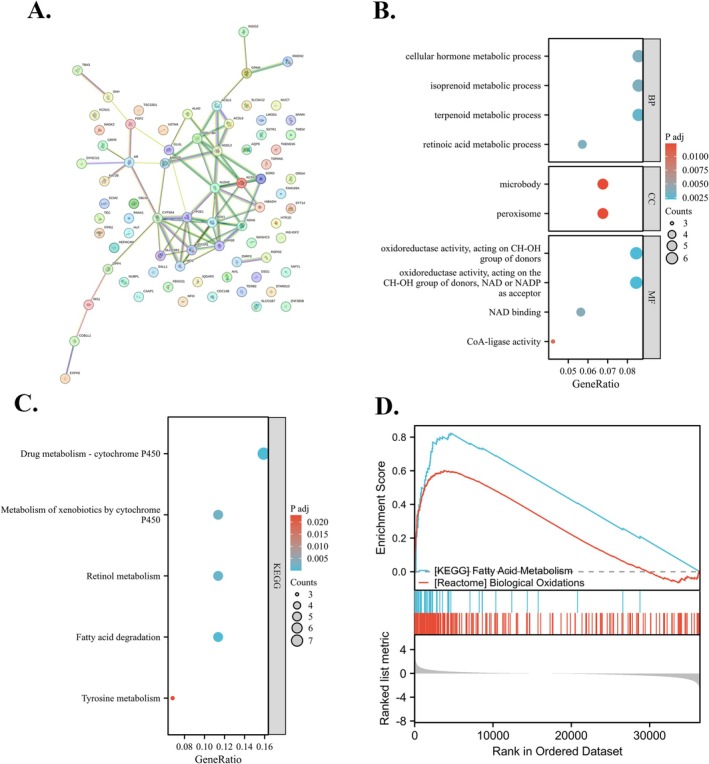
Functional analysis for RANBP3L. (A) PPI network with top‐100 genes that related to RANBP3L. (B) Go enrichment analysis. (C) KEGG pathways analysis. (D) GSEA of RANBP3L in LIHC.

The top 100 genes associated with the RANBP3L gene were presented as a PPI network in Figure 8A. According to the 100 genes, GO enrichment analysis was conducted including BP analysis, CC analysis and MF analysis (Figure 8B). The BP analysis indicated that RANBP3L might play roles in the process of cellular hormone metabolic, isoprenoid metabolic, terpenoid metabolic and retinoic acid metabolic. The CC analysis indicated that RANBP3L might play roles in microbody and peroxisome. The MF analysis suggested that RANBP3L could be involved in functions such as oxidoreductase activity (utilizing NAD or NADP as acceptors), oxidoreductase activity on the CH‐OH group of donors, CoA ligase activity and NAD binding. Based on the top 100 genes, KEGG analysis showed enrichment in the process of fatty acid degradation, metabolism of xenobiotics by cytochrome P450, drug metabolism—cytochrome P450 and retinol metabolism (Figure 8C). Furthermore, the potential functions of RANBP3L in LIHC were explored via the GSEA. The result showed that RANBP3L might play roles in “Fatty Acid Metabolism” and “Biological Oxidations” (Figure 8D).

### 3.7 The Expression and Survival of RANBP3L in LIHC Patients

**FIGURE 9:**
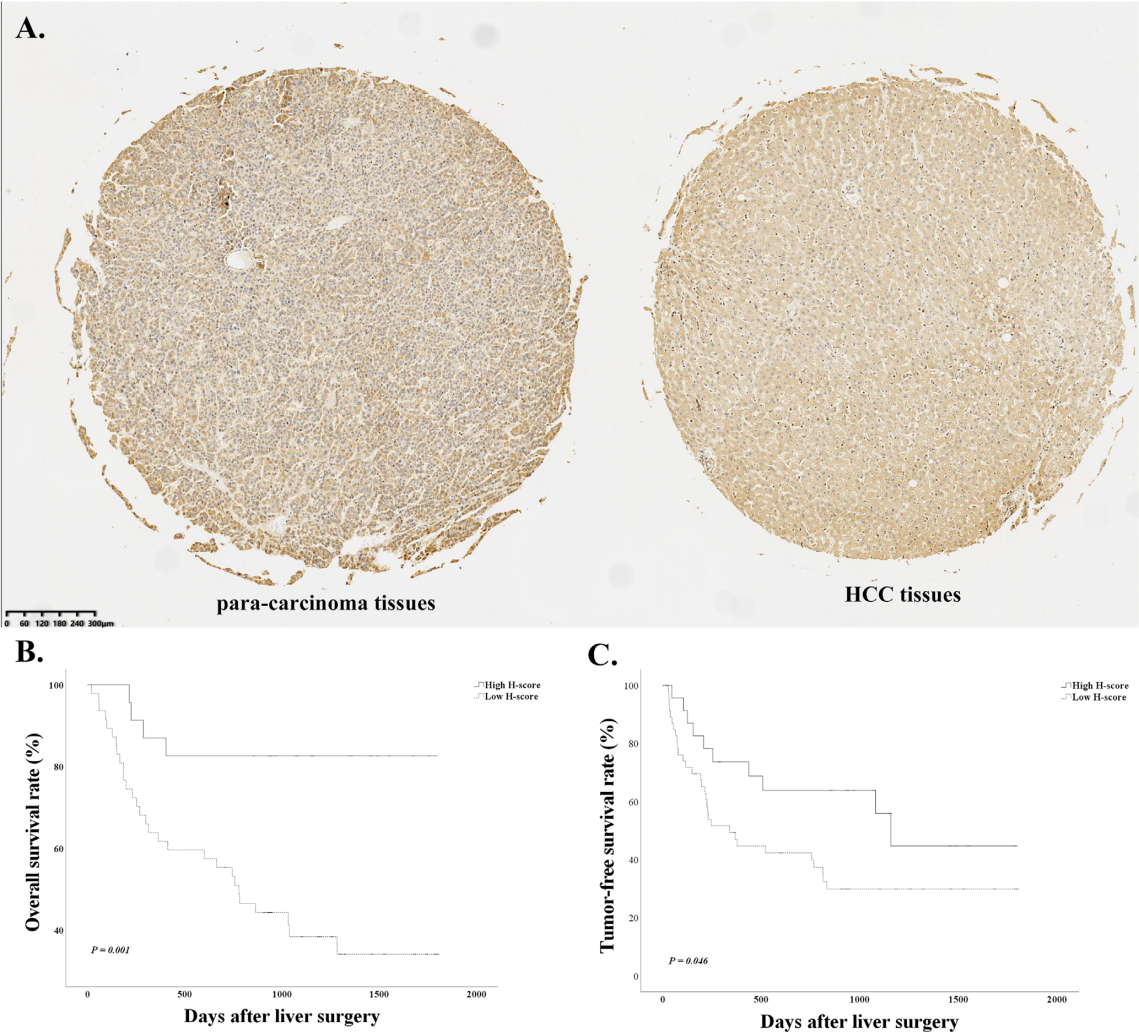
Immunohistochemistry and survival analysis of RANBP3L. (A) Immunohistochemistry analysis of RANBP3L. The expression of para‐carcinoma tissue is higher than LIHC tissue. (B) the overall survival (OS) of LIHC patients. The OS of LIHC patients with high RANBP3L expression is longer than patients with low expression. (C) the tumor‐free survival (TFS) of LIHC patients. The TFS of LIHC patients with high RANBP3L expression is longer than patients with low expression.

**FIGURE 10:**
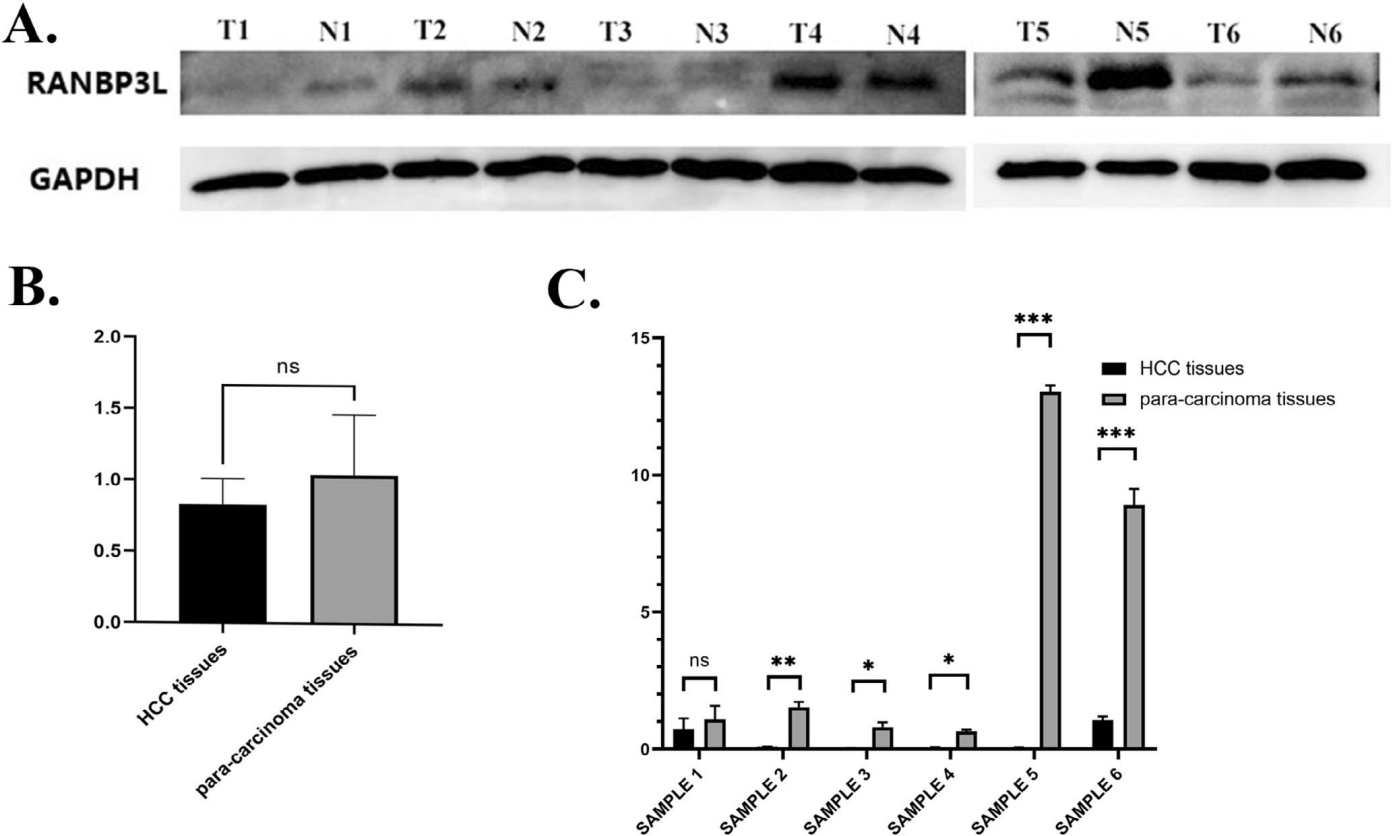
The protein and mRNA expression of RANBP3L in LIHC patients. (A) the protein expression of RANBP3L in LIHC patients, the expression of para‐carcinoma tissue is higher than LIHC tissue. (B) Gray value of western blot, the expression of para‐carcinoma tissue is higher than LIHC tissue without significance. (C) the mRNA expression of RANBP3L in LIHC patients. *p < 0.05, **p < 0.01, ***p < 0.001.

Immunohistochemistry was performed on a tissue microarray comprising 70 LIHC specimens. We used 235 as the cutoff value of H‐score to distinguish high and low expression of RANBP3L. RANBP3L expression was elevated in para‐carcinoma tissues in comparison with LIHC tissues (Figure 9A). Higher RANBP3L expression in LIHC tissues indicated better OS and TFS for LIHC patients (Figure 9B,C). Furthermore, both mRNA and protein levels of para‐carcinoma tissues were higher than LIHC tissues in another 6 LIHC patients (Figure 10).

## 4 Discussion

LIHC is still a leading cancer‐related mortality globally, and the development of effective biomarkers for diagnosis and prognosis should be an area of intense research [3]. We analyzed the expression trends and prognostic relevance of RANBP3L across multiple cancers, particularly focusing on LIHC to find the role of RANBP3L in the LIHC progress.

We got the RANBP3L is corrected with LIHC clinical characteristics such as pathologic M stage, tumor status, AFP (ng/ml), and histologic grade. Further research found the foundation of a prognostic model incorporating RANBP3L expression and clinical features demonstrated its predictive accuracy for survival outcomes in LIHC. The nomogram with LIHC clinical characteristics provides a promising tool for individualized prognosis prediction, and this reduction correlates with poor OS and TFS in LIHC patients. We found RANBP3L is linked to important biological pathways such as oxidoreductase activity and attend cytochrome metabolism which may be regulated by phosphatidylinositol 3 kinase (PI3K)/Akt, EMT, or TGF‐β signaling pathways [15]. Furthermore, this study is the first research to explore the RANBP3L protein and mRNA in clinical LIHC samples. The results showed that RANBP3L might function as a candidate tumor suppressor and the function of tumor suppressor may be achieved through regulating BMP‐driven lineage‐specific differentiation of mesenchymal stem cells [8].

Immunotherapy of LIHC is attracted more and more attention and become very important in the LIHC treatment in recent years [16, 17]. In this research, we found the link between RANBP3L and immune cell infiltration. Immune environment signature parameters can assess prognosis in some kind of cancer types [18], including LIHC [19]. Tregs (CD4^+^CD25^+^Foxp3^+^) representing the most abundant suppressive immune subset in LIHC, and strongly expressing immune checkpoint molecules such as CTLA‐4 and PD‐1, make them direct targets of immune checkpoint inhibitor (ICI) therapy [20]. RANBP3L functions as a factor for phosphorylated SMAD1/5/8 in the BMP pathway, while related RANBP3L regulates SMAD2/3 in the TGF‐β axis they're critical for Treg induction and stability [8]. Recent reviews show that combining TKIs with ICIs has become a frontline strategy in advanced LIHC [21]. RANBP3L exports BMP‐SMADs to terminate BMP signaling, while related RANBP3L modulates TGF‐β/SMAD2/3—pathways known to control FOXP3 induction and Treg stability. Retrospective analyses of ICI ± TKI‐treated cohorts correlating RANBP3L and Treg markers can be considered to assess its predictive and therapeutic potential. These parameters can be used as prognostic elements after immunotherapy [22]. The infiltration of immune cells has been shown to significantly affect LIHC prognosis, assuming its possible regulating LIHC development by the tumor microenvironment remodeling [23]. Those evidences suggests that RANBP3L may affect not only Treg but also other immunosuppressive populations, such as MDSCs and TAMs. These cells contribute to tumor progression and remodeling the extracellular matrix. Investigating RANBP3L's influence on immunity networks could find novel therapeutic strategies.

However, our research has several limitations. Firstly, this study mainly depends on retrospective cohorts and databases, and further validation is required. Secondly, the mechanistic pathways about RANBP3L influence immune cell infiltration and LIHC progression are still unclear and need further investigation through more basic experiments.

## 5 Conclusion

The RANBP3L expression was lower in multiple cancer tissues relative to the normal tissues and significantly associated with the LIHC patients' OS according to the TCGA database. The ROC curve and nomogram model according to the expression of RANBP3L in LIHC could be a good diagnostic indicator. Analysis of LIHC samples revealed that patients exhibiting high RANBP3L expression showed a better prognosis compared to those with low expression. This research found RANBP3L may provide a suspected therapeutic target and predictive biomarker of LIHC.

## Author Contributions

The majority of substantive work during the revision including manuscript drafting and key scientific revisions was taken by Beini Cen. This research was designed and the article was revised by Jie Li. The main manuscript text was written and figures were prepared by Chao Wang. All authors reviewed the manuscript.

## Funding

This work was supported by the Basic Public Welfare Research Plan of Zhejiang Province (LQ24H030009, LTGY24H160023).

## Consent

The authors have nothing to report.

## Conflicts of Interest

The authors declare no conflicts of interest.

## Data Availability

The data that support the findings of this study are openly available in TCGA, UCSC, and TIMER 2.0 databases at DOI: 10.1016/j.cell.2017.05.046, DOI: 10.1038/nmeth.3862 and DOI: 10.1038/s41467‐020‐20217‐2.
